# Clinical significance of skip lymph-node metastasis in pN1 gastric-cancer patients after curative surgery

**DOI:** 10.1093/gastro/goz008

**Published:** 2019-03-11

**Authors:** Jin-Yuan Liu, Jing-Yu Deng, Nan-Nan Zhang, Hui-Fang Liu, Wei-Lin Sun, Wen-Ting He, Yan Wang, Li Zhang, Han Liang

**Affiliations:** 1 Department of Gastroenterology, Tianjin Medical University Cancer Hospital, City Key Laboratory of Tianjin Cancer Center and National Clinical Research Center for Cancer, Tianjin, P. R. China; 2 Department of General Surgery, The Affiliated Hospital of Nankai University, Tianjin, P. R. China

**Keywords:** Stomach, neoplasm, metastasis, prognosis, multivariate analysis

## Abstract

**Background:**

In addition to the stepwise manner of lymph-node metastasis from the primary tumour, the skip lymph-node metastasis (SLNM) was identified as a low-incidence metastasis of gastric cancer (GC). So far, both the mechanism and outcome of SLNM have not been elucidated completely. The purpose of this study was to analyse the clinical significance and the potential mechanism of SLNM in GC patients who had lymph-node metastasis.

**Methods:**

Clinicopathological data and follow-up information of 505 GC patients who had lymph-node metastasis were analysed to demonstrate the significance of SLNM in evaluating the prognostic outcome. According to the pathological results, all GC patients who had lymph-node metastasis were categorized into three groups: patients with the perigastric lymph-node metastasis, patients with the perigastric and extragastric lymph-node metastasis and patients with SLNM.

Results: Among the 505 GC patients who had lymph-node metastasis, 24 (4.8%) had pathologically identified SLNM. The location of lymph-node metastasis was not significantly associated with 5-year survival rate and overall survival (OS) (*P* = 0.194). The stratified survival analysis results showed that the status of SLNM was significantly associated with the OS in patients with pN1 GC (*P* = 0.001). The median OS was significantly shorter in 19 pN1 GC patients with SLNM than in 100 patients with perigastric lymph-node metastasis (*P* < 0.001). The case–control matched logistic regression analysis results showed that tumour size (*P* = 0.002) was the only clinicopathological factor that may predict SLNM in pN1 GC patients undergoing curative surgery. Among the 19 pN1 GC patients with SLNM, 17 (89.5%) had metastatic lymph nodes along the common hepatic artery, around the celiac artery or in the hepatoduodenal ligament.

**Conclusions:**

SLNM may be considered a potentially practicable indicator for prognosis among various subgroups of pN1 GC patients.

## Introduction

Gastric cancer (GC) is the second leading cause of cancer-related deaths worldwide. Moreover, lymph-node metastasis, which represents cancer-cell biological behaviour, has been identified as one of most important clinicopathological variables for evaluating the prognosis of GC patients [1, 2]. The Union for International Cancer Control (UICC) pathological N (pN) category based on the number of metastatic lymph nodes has been generally recognized as the optimal category of lymph-node metastasis for predicting the overall survival (OS) of patients [3]. Some studies insisted that the location of lymph-node metastasis affected the OS independently and showed that the extended lymphadenectomy was not significantly associated with an increase in post-operative death rates [4].

The mechanism of lymph-node metastasis is a sophisticated invasive process throughout the course of GC, and this process covers many kinds of biological behaviours of cancer cells. Referring to the anatomic regions of lymphatic drainage surrounding the stomach, the perigastric lymph nodes should be considered the first-tier lymph nodes that are prone to invasion by cancer cells departing from the primary tumour. The second-tier lymph nodes surrounding the stomach are called extragastric lymph nodes, which usually are located in the original portion of the celiac artery, anterior portion of the common hepatic artery, near half portion of the splenic artery and lower left portion of the hepatoduodenal ligament [5, 6]. In theory, in most GC patients, the spreading cancer cells follow the regular pattern from the first-tier lymph nodes to the second-tier lymph nodes. Actually, a few GC patients without the perigastric lymph-node metastasis would be identified to have the extragastric lymph-node involvement through pathologic examination after surgery, which was called as skip lymph-node metastasis (SLNM) [7]. So far, few investigations focusing on SLNM of cancer have elucidated its clinical significance and its potential mechanism for the purpose of evaluating prognosis [7, 8]. The SLNM distribution of GC patients has not been fully elucidated, although the SLNM occurrence probability of GC has been reported to reach 11% [9]. Some authors have reported that patients with SLNM presented with similar clinicopathological variables and prognosis to those with perigastric lymph-node metastasis and a longer median OS than patients with the perigastric + extragastric lymph-node metastasis after surgery [10]. Other researchers found that the prognosis for patients with SLNM was worse than that of those with the perigastric lymph-node metastasis, and was similar to that of patients with the perigastric and extragastric lymph-node metastases [11].

In this study, we aimed to retrospectively analyse the clinicopathological characteristics of 505 GC patients with lymph-node metastasis to explore the clinical significance of SLNM of GC and the potential mechanism of SLNM in GC patients.

## Methods

### Patients

A total of 1156 patients were diagnosed with gastric adenocarcinoma and underwent the curative gastrectomy plus D2 lymphadenectomy in Tianjin Medical University Cancer Hospital (China) between 2003 and 2011. Eligibility criteria for inclusion in this study were as follows: (i) gastric adenocarcinoma identified by histopathological examination, (ii) histologically confirmed R0 resection, (iii) availability of complete follow-up data, (iv) radical resection and D2 lymphadenectomy performed and (v) no fewer than 16 lymph nodes examined.

### Clinicopathological variables

Medical records were reviewed and the following clinicopathological characteristics were analysed: age at the time of surgery (65 years or younger vs older than 65 years), sex (male vs female), location of the primary tumour (the lower, middle or upper thirds of the stomach vs more than two-thirds of the stomach), size of the primary tumour (4 cm or less vs more than 4 cm), depth of the primary tumour invasion (pT1 vs pT2 vs pT3 vs pT4), Lauren classification (intestinal or diffuse vs mixed), number of metastatic lymph nodes (pN0 vs pN1 vs pN2 vs pN3a vs pN3b), type of gastrectomy (subtotal gastrectomy vs total gastrectomy) and number of examined lymph nodes (fewer than 16 vs 16 or more).

### Follow-up

After curative surgery, all patients were followed every 6 months for the first 2 years and then once a year until death. B-ultrasonography, computed tomography, chest X-ray and endoscopy were performed every visit.

### Statistical analysis

The median OS was determined using the Kaplan–Meier method. The log-rank test was used to compare the survival distributions of each univariate. The variables that were deemed to be of potential importance in univariate analyses (*P* < 0.05) were included in the multivariate analyses. Multivariate analyses were performed by means of the Cox proportional hazards model, using the forward stepwise procedure for variable selection. Hazard ratios (HRs) and 95% confidential intervals (CIs) were generated. To assess the potential bias in comparing prognostic factors with different clinicopathological characteristics, the Bayesian Information Criterion (BIC) was used. A smaller BIC value indicated a better model for predicting outcome. To overcome the constituent ratio error among the subpopulation of patients, case–control matched logistic regression was used. Chi-square was adopted to demonstrate the association between SLNM and various clinicopathological variables in the logistic regression analyses. The significance level was defined as *P* < 0.05. All statistical analyses were performed using a statistical analysis program package SPSS 22.0 (SPSS Inc; Chicago, IL, USA).

## Results

### Clinicopathological outcomes

In the present retrospective study, data from 505 consecutive patients (363 males and 142 females) with lymph-node metastasis for primary GC between March 2003 and August 2011 were examined. The median follow-up period was 84 months (range, 6–144 months). The patients’ ages ranged from 20 to 87 years, with an average age of 59.1 years. In accordance with the 8th edition of the UICC/American Joint Committee on Cancer (AJCC) pathological TNM classification of GC, of the 505 patients, 125 (15.9%), 183 (23.1%), 138 (17.4%) and 59 (7.4%) had pN1, pN2, pN3a and pN3b category GC, respectively (Supplementary Table 1). The type of gastrectomy (total gastrectomy for 178 patients and subtotal gastrectomy for 237 patients) was selected based mainly on the GC treatment guidelines in Japan. Among 505 patients with lymph-node metastasis, 275 had perigastric lymph-node metastasis, 206 had perigastric + extragastric lymph-node metastasis and 24 had SLNM. The 5-year survival rate of the patients with lymph-node metastasis was 19.0%; 96 patients were alive at the last follow-up and the median OS of all patients after surgery was 25.0 months.

### Univariate survival analysis

**Figure 1.**
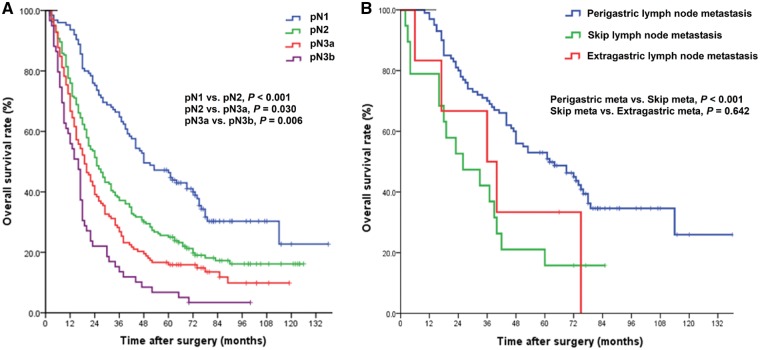
Survival curves for gastric-cancer (GC) patients with lymph-node metastasis. (**A**) Comparison of survivals between subgroups of different N categories (based on the 8th Union for International Cancer Control pathological TNM classification of GC) among 505 GC patients. (**B**) Comparison of survival between subgroups of different locations of metastatic lymph nodes among 125 pN1 GC patients. Perigastric meta, perigastric lymph-node metastasis; Skip meta, skip lymph-node metastasis; Extragastric meta, extragastric lymph-node metastasis.

The univariate analysis showed that, in GC patients with lymph-node metastasis, age at surgery (*P* = 0.021), tumour size (*P* = 0.005), type of gastrectomy (*P* = 0.001), Lauren’s classification of primary tumour (*P* = 0.050), depth of primary tumour invasion (pT category) (*P* < 0.001), location of lymph-node metastasis (*P* < 0.001) and number of metastatic lymph nodes (pN category) (*P* < 0.001) were significantly associated with the median OS of patients (Supplementary Table 1). We found that (i) the more deeply the primary tumour invaded, the shorter the median OS of patients was; (ii) the higher the number of metastatic lymph nodes, the shorter the median OS of patients was; and (iii) the median OS of patients with extragastric lymph-node metastasis was shorter than that of patients with perigastric lymph-node metastasis or that of patients with SLNM (Figure 1A).

### Multivariate survival analysis

All night variables listed above were included in a multivariate Cox proportional hazards model (forward stepwise procedure) to adjust for the effects of covariates (Supplementary Table 1). In that model, age at surgery (*P* = 0.006), depth of primary tumour invasion (*P* = 0.002) and number of metastatic lymph nodes (*P* < 0.001) were significantly associated with the median OS of GC patients with lymph-node metastasis. However, the location of metastatic lymph nodes was not significantly associated with the median OS of GC patients with lymph-node metastasis (*P* = 0.194). Thus, we analysed the median OS of patients with SLNM on respective pN stages. By using the stratified survival analysis, we found that SLNM was significantly associated with the median OS in patients with pN1 GC (perigastric lymph-node metastasis vs SLNM, *P* < 0.001; Supplementary Table 2).

### Univariate and multivariate survival analyses of pN1 GC patients

The univariate analyses showed that the type of gastrectomy (*P* = 0.031) and the location of metastatic lymph nodes (*P* = 0.002) were significantly associated with the median OS of pN1 GC patients (Table 1). We included the two variables mentioned above into a multivariate Cox proportional hazards model (forward stepwise procedure) to adjust for the effects of covariates. The results showed that the location of metastatic lymph nodes (HR = 1.675; 95% CI = 1.184–2.370, *P* = 0.004) and type of gastrectomy (HR = 1.624; 95% CI = 1.022–2.581, *P* = 0.040) were significantly associated with the median OS of pN1 GC patients (Table 1).

**Table 1.** Univariate and multivariate survival analyses of 125 pN1 gastric-cancer patients undergoing curative gastrectomy

Clinicopathological characteristic	No. of cases	5-year survival rate (%)		Median OS (months)	*χ* ^2^	Univariate *P*-value	HR	95% CI	BIC value	Multivariate *P*-value
Sex					0.237	0.627				
Male	87	35.6		48.0						
Female	38	28.9		44.0						
Age at surgery					1.477	0.224				
≤65 years	2	0.0		12.0						
>65 years	123	34.1		48.0						
Tumour size					2.135	0.144				
≤4 cm	52	40.4		69.0						
>4 cm	73	28.8		44.0						
Tumour location					9.143	0.270				
Upper third	33	27.3		46.0						
Middle third	8	0.0		24.0						
Lower third	60	38.3		69.0						
More than two-thirds stomach	24	41.7		39.0						
Lauren classification					1.117	0.291				
Intestinal	42	40.5		62.0						
Diffuse	78	32.1		48.0						
Mixed	0	–								
Depth of primary tumour invasion (pT category)					5.479	0.242				
pT1	2	100.0		59.0						
pT2	17	52.9		60.0						
pT3	9	22.2		48.0						
pT4a	96	30.2		42.0						
pT4b	1	0.0		60.0						
Location of metastatic lymph nodes					12.355	0.002	1.675	1.184–2.370	28.683	0.004
Perigastric LNM	100	38.0		62.0						
SLNM	19	15.8		26.0						
Perigastric + extragastric LNM	6	16.7		36.0						
Number of lymph nodes examinedl					0.061	0.805				
≤16	59	32.2		48.0						
>16	66	34.8		48.0						
Type of gastrectomy					4.646	0.031	1.624	1.022–2.581	32.467	0.040
Subtotal	91	38.5		69.0						
Total	34	20.6		39.0						

OS, overall survival; HR, hazard ration; CI, confidential interval; BIC, Bayesian Information Criterion; LNM, lymph-node metastasis; SLNM, skip lymph-node metastasis.

The median OS was longer in GC patients who underwent subtotal gastrectomy than in those who underwent total gastrectomy (69.0 vs 39.0 months, log-rank *P* = 0.031); the median OS of patients with SLNM was shorter than that of those with perigastric lymph-node metastasis (26.0 vs 62.0 months, *P* < 0.001); however, there was no statistical difference between the median OS of patients with SLNM and perigastric + extragastric lymph-node metastases (26.0 vs 36.0 months, *P* = 0.642; Figure 1B).

### BIC value performance

BIC values were obtained by using logistic regression according to the survival status of patients. We found that the BIC value of the location of metastatic lymph nodes was lower than that of the type of gastrectomy (28.683 vs 32.467) in patients with pN1 GC (Table 1).

### Associations between SLNM and various clinicopathological variables in pN1 GC patients based on the case–control matched logistic regression

We adopted case–control matched logistic regression (using the forward stepwise procedure) to directly analyse the various clinicopathological variables and considered the different statuses of SLNM. We matched 125 patients in terms of sex (male vs female), age at surgery, tumour size, tumour location (lower third vs middle third vs upper third vs more than two-thirds of the stomach), depth of primary tumour invasion (pT1 vs pT2 vs pT3 vs pT4), Lauren classification of primary tumour (intestinal vs diffuse vs mixed), number of lymph nodes and type of gastrectomy (subtotal vs total). The results showed that the tumour size (*P* = 0.002, χ^2^^ ^= 30.476) was the only clinicopathological variable associated with the SLNM in pN1 GC patients undergoing curative surgery (*P* = 0.002, χ^2^^ ^= 30.476; Table 2). Among the pN1 GC patients with SLNM, 17 (89.5%) had positive lymph nodes along the common hepatic artery, around the coeliac artery or in the hepatoduodenal ligament (Supplementary Table 3).

**Table 2.** Association between SLNM and various clinicopathological variables in pN1 gastric-cancer patients

Clinicopathological characteristic	SLNM		*χ* ^2^	Univariate *P*-value
	Yes (*n* = 19)	No (*n* = 106)		
Sex			1.451	0.228
Male	11 (57.9)	76 (71.7)		
Female	8 (42.1)	30 (28.3)		
Age at surgery (years)^a^	53.4 ± 12.0	59.8 ± 11.8	0.364	0.127
Tumour size (cm)^a^	7.9 ± 5.8	5.289 ± 2.3	30.476	0.002
Location			4.501	0.212
Upper third	4 (21.1)	29 (27.4)		
Middle third	1 (5.3)	7 (6.6)		
Lower third	7 (36.8)	53 (50.0)		
More than two-thirds of the stomach	7 (36.8)	17 (16.0)		
Lauren classification			0.026	0.871
Intestinal	7 (36.8)	37 (34.9)		
Diffuse	12 (63.2)	69 (65.1)		
Mixed	0 (0)	0 (0)		
Depth of primary tumour invasion (pT category)			2.775	0.596
pT1	0 (0)	2 (1.9)		
pT2	2 (10.5)	15 (14.2)		
pT3	0 (0)	9 (8.5)		
pT4a	17 (89.5)	79 (74.5)		
pT4b	0 (0)	1 (0.9)		
Number of lymph nodes examined^a^	21.1 ± 13.5	18.2 ± 11.0	34.851	0.388
Type of gastrectomy			1.052	0.305
Subtotal	12 (63.2)	79 (74.5)		
Total	7 (36.8)	27 (25.5)		

^a^These values are presented as mean ± standard deviation; other values are expressed as number of patients followed by percentage in parentheses.

SLNM, skip lymph-node metastasis.

## Discussion

The lymph-node metastasis from GC basically follows the law of stepwise spread through the anatomical regional lymphatic bed; however, predicting the second-tier lymph-node metastasis, including the SLNM, is impossible. Therefore, D2 lymphadenectomy is recommended as the key procedure in curative gastrectomy, even for some early-stage GC patients with suspected lymph-node metastasis [7, 12, 13]. Some studies suggested that minimally invasive therapy of lymph-node dissection should be supplemented for early-stage GC patients who undergo endoscopic mucosal resection, wedge resection or laparoscopy-assisted gastrectomy, taking into consideration the potential of lymph-node metastases and SLNM [14, 15]. Kim *et al.* [7] analysed the data of 997 GC patients with lymph-node metastasis and found that patients with SLNM showed a lower frequency of vascular invasion than those with first-tier lymph-node metastasis, and showed smaller tumour size and lower incidence of lymphatic, vascular and perineural invasions than those with the stepwise second-tier lymph-node metastasis. Moreover, researchers have agreed that predicting whether patients had SLNM before surgery is impossible at this point; therefore, D2 lymphadenectomy is recommended as the optimal treatment strategy for patients with potential SLNM.

Theoretically, the SLNM of GC can be presented in the following situations: (i) true SLNM, which may be induced from the blockage of afferent lymphatic vessels among partial first-tier lymph nodes [16] and (ii) false SLNM, which indicates that cancer cells have invaded the extragastric lymph nodes gradually through the local lymphatic vessels; however, the perigastric lymph-node metastasis cannot be examined because of the morphological and structural damage in the first-tier lymph nodes by cancer-cell proliferation [17], only micro-metastases or isolated tumour cells in the first-tier lymph nodes [18] or the insufficiently examined lymph-node count [19]. In these studies, 24 (4.7%) of 505 GC patients were pathologically identified as SLNM cases after curative gastrectomy with D2 lymphadenectomy. The median OS of those patients with SLNM was 23.0 months, which is shorter than that of patients with the perigastric lymph-node metastasis (32.0 months) and higher than that of patients with perigastric + extragastric lymph-node metastasis (19.0 months). No statistically significant difference in the median OS was observed between patients with SLNM, patients with the perigastric lymph-node metastasis and patients with the perigastric + extragastric lymph-node metastasis in this study. Therefore, we considered that SLNM should be deemed as the potential perigastric + extragastric lymph-node metastasis in terms of patient prognosis and pathological outcomes. Furthermore, we found that SLNM is only applicable for distinguishing the differences in median OS among subgroups of pN1 stage GC patients by using the stratified survival analysis (Supplementary Table 2). Upon multivariate survival analysis, the location of metastatic lymph nodes (*P* = 0.004) and the type of gastrectomy (*P* = 0.040) were identified as the independent predictors for evaluating the median OS of pN1 stage patients after curative surgery (Table 1). Among those independent prognostic predictors, the locations of metastatic lymph nodes were demonstrated as the most intensive factor when evaluating the prognosis of pN1 stage patients after curative surgery, owing to the low BIC value of SLNM (Table 1). Additionally, the logistic regression analysis between SLNM and other clinicopathological variables showed that only the tumour size was a relative factor to the SLNM in pN1 stage patients in this study. Compared with previous reports, our study included a higher proportion of advanced GC patients (98.4%) and presented a comparatively shorter OS. Therefore, we do think that many patients with SLNM in this study might be false SLNM patients whose perigastric lymph nodes were destroyed by cancer cells or that the examined lymph-node counts were not enough.

In conclusion, the causes of SLNM of GC remain ambiguous and vague in clinical settings. The pN1 stage GC patients with SLNM presented a worse prognosis than those without SLNM. The examined lymph-node count, based on standard lymph-node dissection (D2 lymphadenectomy), should be sufficient for improving the accuracy of SLNM in GC patients after surgery. Tumour size, as an important relative factor to SLNM prediction, indicated that neoadjuvant chemotherapy needs to be recommended for patients with large GC tumours for eradicating the micro-metastasis in the lymphatic system [20].

## Authors’ contributions

J.Y.L., J.Y.D. and H.L. conceived of the study and participated in its design. J.Y.L. and J.Y.D. performed the statistical analyses and interpretation. J.Y.L., J.Y.D. and N.N.Z. drafted the manuscript. All authors read and approved the final manuscript.

## Funding

This study was supported in part by grants from the Programs of National Natural Science Foundation of China (No. 81572372), the National Key Research and Development Program ‘Major Chronic Non-Infectious Disease Research’ of China (No. 2016YFC1303202) and the National Key Research and Development Program ‘Precision Medicine Research’ of China (No. 2017YFC0908304).

## Supplementary Material

goz008_Supplementary_Data

## References

[1] Maruyama K The most important prognostic factors for gastric cancer patients: a study using univariate and multivariate analyses. Scand J Gastroenterol (Suppl) 2009;22:63–8.

[2] Takagane A, Terashima M, Abe K et al Evaluation of the ratio of lymph node metastasis as a prognostic factor in patients with gastric cancer. Gastric Cancer 1999;2:122–8. 11957084 10.1007/s101200050034

[3] Sano T, Coit DG, Kim HH et al Proposal of a new stage grouping of gastric cancer for TNM classification: International Gastric Cancer Association staging project. Gastric Cancer 2017;20:217–25. 26897166 10.1007/s10120-016-0601-9 PMC4992472

[4] De Manzoni G, Verlato G, Guglielmi A et al Prognostic significance of lymph node dissection in gastric cancer. Br J Surg 1996;83:1604–7. 9014687 10.1002/bjs.1800831137

[5] Nakajima T Gastric cancer treatment guidelines in Japan. Gastric Cancer 2002;5:1–5. 10.1007/s101200200000 12021853

[6] In H, Ravetch E, Langdon-Embry M et al The newly proposed clinical and post-neoadjuvant treatment staging classifications for gastric adenocarcinoma for the American Joint Committee on Cancer (AJCC) staging. Gastric Cancer 2018;21:1–9. 28948368 10.1007/s10120-017-0765-y

[7] Kim DH, Choi MG, Noh JH et al Clinical significance of skip lymph node metastasis in gastric cancer patients. Eur J Surg Oncol 2015;41:339–45. 25454830 10.1016/j.ejso.2014.09.009

[8] Merrie AE, Phillips LV, Yun K et al Skip metastases in colon cancer: assessment by lymph node mapping using molecular detection. Surgery 2001;129:684–91. 11391366 10.1067/msy.2001.113887

[9] Kosaka T, Ueshige N, Sugaya J et al Lymphatic routes of the stomach demonstrated by gastric carcinomas with solitary lymph node metastasis. Surg Today 1999;29:695–700. 10483741 10.1007/BF02482311

[10] Saito H, Tsujitani S, Ikeguchi M Clinical significance of skip metastasis in patients with gastric cancer. Gastric Cancer 2007;10:87–91. 17577617 10.1007/s10120-007-0412-0

[11] Choi YY, An JY, Guner A et al Skip lymph node metastasis in gastric cancer: is it skipping or skipped? Gastric Cancer 2016;19:206–15. 25708370 10.1007/s10120-015-0472-5

[12] Sano T, Hollowood A Early gastric cancer: diagnosis and less invasive treatments. Scand J Surg 2006;95:249–55. 17249273 10.1177/145749690609500407

[13] Degiuli M, Sasako M, Ponti A et al Randomized clinical trial comparing survival after D1 or D2 gastrectomy for gastric cancer. Br J Surg 2014;101:31–2. 24375296 10.1002/bjs.9345

[14] Sang EL, Lee JH, Ryu KW et al Sentinel node mapping and skip metastases in patients with early gastric cancer. Ann Surg Oncol 2009;16:603–8. 19127361 10.1245/s10434-008-0283-6

[15] Soetikno R, Kaltenbach T, Yeh R et al Endoscopic mucosal resection for early cancers of the upper gastrointestinal tract. JCO 2005;23:4490–8. 10.1200/JCO.2005.19.935 16002839

[16] Hoshi H, Kamiya K, Aijima H et al Histological observations on rat popliteal lymph nodes after blockage of their afferent lymphatics. Arch Histol Jpn 1985;48:135–48. 3899053 10.1679/aohc.48.135

[17] Jiangtao G, Yi P, Xiaofan G et al Effect of the number of positive niduses in extranodal soft tissues on the overall survival of gastric cancer patients. Int J Clin Exp Pathol 2017;10:11090–7. PMC6965835 31966457

[18] Jeuck TL, Wittekind C Gastric carcinoma: stage migration by immunohistochemically detected lymph node micrometastases. Gastric Cancer 2015;18:100–8. 24550066 10.1007/s10120-014-0352-4

[19] Deng J, Yamashita H, Seto Y et al Increasing the number of examined lymph nodes is a prerequisite for improvement in the accurate evaluation of overall survival of node-negative gastric cancer patients. Ann Surg Oncol 2017;24:745–53. 27770340 10.1245/s10434-016-5513-8

[20] Zhenhui L, Dafu Z, Youguo D et al Computed tomography-based radiomics for prediction of neoadjuvant chemotherapy outcomes in locally advanced gastric cancer: a pilot study. Chin J Cancer Res 2018;30:406–14. 30210220 10.21147/j.issn.1000-9604.2018.04.03 PMC6129565

