# Supplementary material for: Clinical significance of skip lymph-node metastasis in pN1 gastric-cancer patients after curative surgery
Source: Gastroenterol Rep (Oxf). 2019 Mar 11;7(3):193–8. doi: 10.1093/gastro/goz008 (PMC6573797; doi:10.1093/gastro/goz008)
Supplement: goz008_Supplementary_Data [file goz008_supplementary_data.docx]

**Supplementary Table 1. Clinicopathological characteristics of 505 with lymph node metastasis gastric cancer patients undergoing curative gastrectomy**

| **Clinicopathological**  **characteristic** | **No. of**  **cases** | **5-year survival rate** **(%)** | **Median OS (months)** | **χ^2^** | **Univariate**  ***P* value** | **HR** | **95% CI** | **Multivariate *P* value** |
| --- | --- | --- | --- | --- | --- | --- | --- | --- |
| **Sex** |  |  |  | 0.350 | 0.554 | 0.921 | 0.735-1.156 | 0.479 |
| Male | 363 | 19.0 | 24.0 |  |  |  |  |  |
| Female | 142 | 19.0 | 26.0 |  |  |  |  |  |
| **Age at surgery** |  |  |  | 5.319 | 0.021 | 1.351 | 1.090-1.675 | 0.006 |
| ≤65 years | 344 | 20.6 | 28.0 |  |  |  |  |  |
| >65 years | 161 | 15.5 | 21.0 |  |  |  |  |  |
| **Tumor size** |  |  |  | 7.875 | 0.005 | 1.137 | 0.915-1.413 | 0.246 |
| ≤4 cm | 178 | 25.3 | 29.0 |  |  |  |  |  |
| >4 cm | 327 | 15.6 | 23.0 |  |  |  |  |  |
| **Tumor location** |  |  |  | 4.803 | 0.187 | 0.994 | 0.903-1.093 | 0.897 |
| Upper third | 95 | 22.1 | 34.0 |  |  |  |  |  |
| Middle third | 52 | 9.6 | 19.0 |  |  |  |  |  |
| Lower third | 243 | 18.9 | 27.0 |  |  |  |  |  |
| More than two-thirds stomach | 115 | 20.9 | 22.0 |  |  |  |  |  |
| **Lauren classification** |  |  |  | 5.980 | 0.050 | 1.125 | 0.897-1.412 | 0.308  308 |
| Intestinal | 117 | 23.9 | 34.0 |  |  |  |  |  |
| Diffuse | 377 | 17.8 | 24.0  16 |  |  |  |  |  |
| Mixed | 11 | 9.1 | 34.0 |  |  |  |  |  |
| **Depth of primary tumor invasion (pT category)** | | |  | 29.571 | <0.001 | 1.346 | 1.114-1.625 | 0.002 |
| pT1a | 2 | 100.0 | 65.0 |  |  |  |  |  |
| pT1b | 2 | 50.0 | 59.0 |  |  |  |  |  |
| pT2 | 36 | 41.7 | 51.0 |  |  |  |  |  |
| pT3 | 25 | 28.0 | 50.0 |  |  |  |  |  |
| pT4a | 422 | 16.8 | 24.0 |  |  |  |  |  |
| pT4b | 18 | 0.0 | 9.0 |  |  |  |  |  |
| **Number of metastatic lymph nodes (pN category)** | | |  | 67.027 | <0.001 | 1.416 | 1.251-1.603 | <0.001 |
| pN1 | 125 | 33.6 | 48.0 |  |  |  |  |  |
| pN2 | 183 | 18.6 | 25.0 |  |  |  |  |  |
| pN3a | 138 | 13.0 | 19.0 |  |  |  |  |  |
| pN3b | 57 | 3.4 | 16.0 |  |  |  |  |  |
| **Location of metastatic lymph nodes** | | |  | 25.223 | <0.001 | 1.078 | 0.962-1.208 | 0.194 |
| Perigastric LNM | 275 | 25.5 | 32.0 |  |  |  |  |  |
| SLNM | 24 | 16.7 | 23.0 |  |  |  |  |  |
| Perigastric + extragastric LNM extragastric ric | 206 | 10.7 | 19.0 |  |  |  |  |  |
| **Number of lymph nodes examined** | | |  | 1.299 | 0.254 | 0.908 | 0.732-1.127 | 0.383 |
| ≤16 | 193 | 20.7 | 28.0 |  |  |  |  |  |
| >16 | 312 | 17.9 | 22.0 |  |  |  |  |  |
| **Type of gastrectomy** |  |  |  | 10.910 | 0.001 | 1.181 | 0.962-0.450 | 0.113 |
| Subtotal | 327 | 22.3 | 28.0 |  |  |  |  |  |
| Total | 178 | 12.9 | 20.0 |  |  |  |  |  |

HR, hazard ration; CI, confidential interval; LNM, lymph node metastasis; SLNM, skip lymph node metastasis**.**

**Supplementary Table 2. SLNM was demonstrated to be feasibly applicable to evaluate the prognosis only in pN1 stage patients by using the strata survival analysis**

| **Clinicopathological characteristic** | **No. of cases** | **5-year survival rate (%)** | **Median OS (months)** | **SLNM** | |
| --- | --- | --- | --- | --- | --- |
|  |  |  |  | **χ^2^** | ***P* value** |
| **pN1** |  |  |  |  |  |
| Perigastric LNM | 100 | 38.0 | 62.0 | 11.158 | 0.001 |
| SLNM | 19 | 15.8 | 26.0 |  |  |
| Perigastric + extragastric LNM | 6 | 16.7 | 36.0 | 0.216 | 0.642 |
| **pN2** |  |  |  |  |  |
| Perigastric LNM | 108 | 21.3 | 25.0 | 0.122 | 0.727 |
| SLNM | 5 | 20.0 | 16.0 |  |  |
| Perigastric + extragastric LNM | 70 | 14.3 | 25.0 | 0.001 | 0.981 |

LNM, lymph node metastasis; SLNM, skip lymph node metastasis.

**Supplementary Table 3. Location of skip lymph node metastasis in pN1 gastric cancer patients underwent the curative gastrectomy**

| **No. of patients** | **No. 8 nodal involvement** | **No. 9 nodal involvement** | **No. 10 nodal involvement** | **No. 11 nodal involvement** | **No. 12 nodal involvement** | **Number of extragastric examined nodes** |
| --- | --- | --- | --- | --- | --- | --- |
| **1** | 1 |  |  |  |  | 3 |
| **2** |  | 2 |  |  |  | 5 |
| **3** | 1 |  |  |  |  | 4 |
| **4** | 2 |  |  |  |  | 4 |
| **5** | 1 |  |  |  |  | 7 |
| **6** | 1 | 1 |  |  |  | 3 |
| **7** |  |  |  |  | 2 | 2 |
| **8** | 1 |  |  |  |  | 5 |
| **9** |  |  |  | 1 |  | 9 |
| **10** |  |  |  |  | 1 | 5 |
| **11** |  |  |  |  | 1 | 8 |
| **12** | 1 |  |  |  |  | 2 |
| **13** |  |  |  |  | 1 | 1 |
| **14** |  |  | 1 |  |  | 8 |
| **15** |  |  |  |  | 1 | 10 |
| **16** | 1 |  |  |  |  | 5 |
| **17** |  |  |  |  | 2 | 12 |
| **18** | 1 |  |  |  |  | 10 |
| **19** |  | 2 |  |  |  | 23 |
